# FYN is required for ARHGEF16 to promote proliferation and migration in colon cancer cells

**DOI:** 10.1038/s41419-020-02830-1

**Published:** 2020-08-07

**Authors:** Bei Yu, Linlin Xu, Limin Chen, Yao Wang, Hongying Jiang, Yiting Wang, Yehong Yan, Shiwen Luo, Zhenyu Zhai

**Affiliations:** 1 Center for Experimental Medicine, the First Affiliated Hospital of Nanchang University, Nanchang, Jiangxi China; 2 Jiangxi Key Laboratory of Molecular Diagnostics and Precision Medicine, Nanchang, Jiangxi China; 3 School of Biotechnology and Health Sciences, Wuyi University, Jiangmen Guangdong, China; 4 School of Public Health of Nanchang University, Nanchang, Jiangxi China; 5 Department of General Surgery, the First Affiliated Hospital of Nanchang University, Nanchang, Jiangxi China

**Keywords:** Oncogenes, Gastrointestinal diseases

## Abstract

ARHGEF16 is a recently identified Rho-family guanine nucleotide exchange factor (GEF) that has been implicated in the activation of Rho-family GTPases such as Rho G, Rac, and Cdc42. However, its functions in colon cancer cell proliferation and migration are not well understood. In this study, we showed that ARHGEF16 was highly expressed in clinical specimens of colon cancer. In colon cancer cells, ARHGEF16-stimulated proliferation and migration in vitro and in vivo. Furthermore, we identified a nonreceptor tyrosine kinase, FYN, as a novel partner of ARHGEF16. Knocking down FYN expression decreased ARHGEF16 protein level in colon cancer cells. We further demonstrated that ARHGEF16-induced colon cancer cell proliferation and migration were dependent on FYN since knockdown FYN abolished the ARHGEF16-induced proliferation and migration of colon cancer cells. The FYN-ARHGEF16 axis mediates colon cancer progression and is a potential therapeutic target for colon cancer treatment.

## Introduction

The small GTPases of the Rho family (Rho-family GTPases) have manifold physiological functions, such as roles in cell adhesion, cytoskeleton regulation, cell proliferation and motility, and tumorigenesis^1–3^. RhoA, RhoC, Rac1, and Cdc42 are members of the Rho family of GTPases, and their activation is regulated by transforming the GDP-bound form into the GTP-bound form, while aberrantly active Rho-family GTPases serve as regulators of cellular functions crucial for cancer progression^4,5^. Furthermore, the status of bound GTP is known to be regulated by guanine nucleotide exchange factors (GEFs)^6,7^. Remarkably, GEFs play important signal transduction roles in physiological and oncopathological events by regulating the activity of Rho GTPases.

ARHGEF16 is a GEF that catalyzes the exchange of GDP nucleotide for GTP and plays key roles in the activation of RhoG, Rac1, and Cdc42^8–11^. ARHGEF16 contains a central Dbl homology (DH) domain, a Pleckstrin homology (PH) domain and a C-terminal Src homology-3 (SH3) domain^8^. Recent studies have shown that ARHGEF16 activates Rac1 via the RhoG–Elmo–Dock4 pathway^11^. Yamaki et al.^8^ reported that ARHGEF16 bound to EphA2 and modulated the migration of breast cancer cells in a RhoG-dependent manner. Further studies have shown that ARHGEF16 activating RhoG and PI3K downstream of EphA2 contributes to apoptosis resistance in tumor cells^9,12^. Most remarkably, ARHGEF16 preferentially binds to Elmo1 and plays a critical role in enhancing the engulfment of apoptotic cells^11^. Previous reports from our laboratory have shown that aberrant activation of Gli2, a glioma-associated oncogene and zinc-finger transcription factor for hedgehog signaling, increases the transcript level of ARHGEF16, while ARHGEF16 interacts with cytoskeleton-associated protein 5 (CKAP5) to promote the proliferation and migration of glioma cells^13^. Thus, ARHGEF16 is critical for cancer cell proliferation and growth as well as tumorigenesis. However, it remains unclear whether there are other yet unknown ARHGEF16 signaling pathways that are important in the progression of colon cancer.

To further address this remaining question and elucidate this issue, we identified FYN as a novel partner of ARHGEF16 that can specifically bind to ARHGEF16. FYN is a nonreceptor tyrosine kinase, specifically a Src family kinase (SFK), that plays a critical role in the development and progression of several cancer types by regulating morphogenic transformation, cellular motility, cell growth, and cell death^14–17^. However, what is exactly FYN functions in colon cancer, including whether it works directly with ARHGEF16 to perform an oncogenic function or plays a role in tumorigenesis associated with this oncogenic function, remains unknown.

Our studies detailed below investigated the roles of FYN and ARHGEF16 and their relationship in colon cancer through in vitro experiments. We also showed that ARHGEF16-induced colon cancer cell proliferation and migration were tightly dependent on FYN. Our results suggested that FYN-ARHGEF16 signaling could serve as a novel molecular target for developing anti-colon cancer therapies.

## Results

### ARHGEF16 is highly expressed in colon cancer tissues

**Fig. 1:**
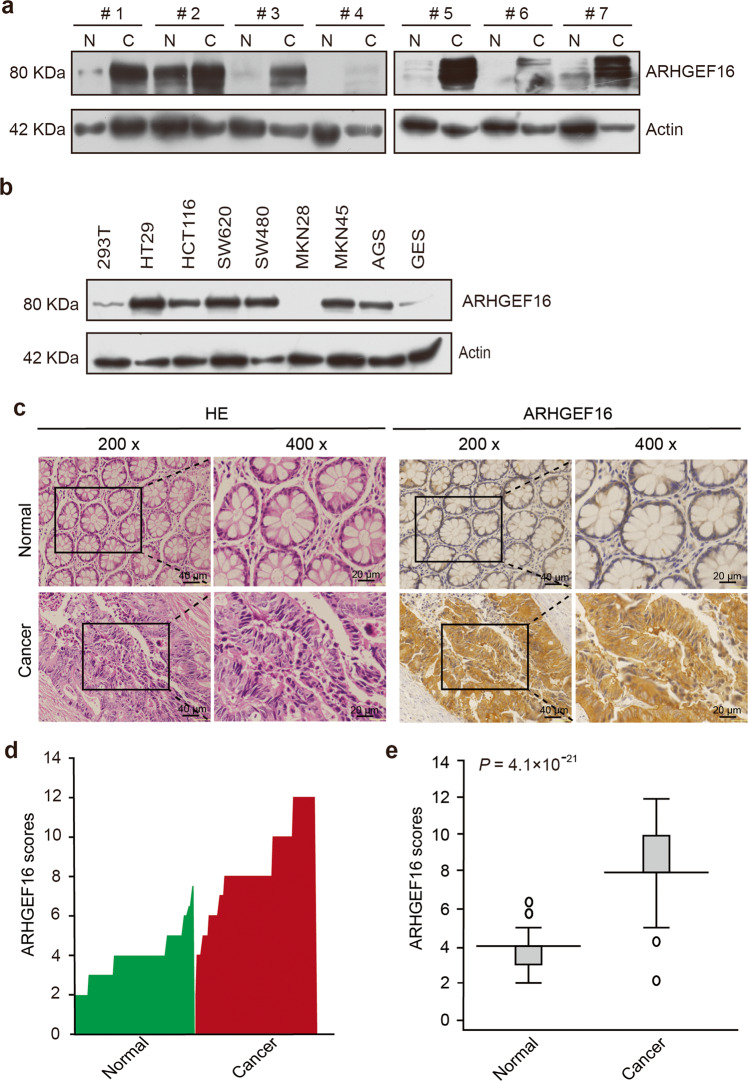
ARHGEF6 expression is elevated in colorectal cancer tissue samples. **a** ARHGEF16 is highly expressed in colorectal cancer tissue samples. Proteins isolated from colon cancer and adjacent nontumorous tissue samples obtained from seven patients were separated by SDS–PAGE and subjected to WB analysis. N normal tissue, T tumor tissue. **b** ARHGEF16 is highly expressed in colorectal cancer cell lines compared with the HEK293T cell line and gastric cancer cell lines as examined by WB analysis. **c** ARHGEF16 is overexpressed in colorectal cancer tissue samples compared with normal colorectal tissue samples as examined by IHC. Representative images are shown. **d** The ARHGEF16 scores of each colorectal cancer and normal colorectal tissue sample were plotted. **e** Box plots of the scores for ARHGEF16 expression are shown. Statistical significance was analyzed using the Mann–Whitney *U* test, *n* = 71.

To investigate the role of ARHGEF16 in colon cancer, we evaluated ARHGEF16 levels in seven paired samples of colon cancer tissue and adjacent normal tissue by WB analysis and revealed that the expression of the ARHGEF16 protein was higher in the colon cancer tissue samples than in the normal tissue samples (Fig. 1a). The ARHGEF16 protein expression was also higher in colon cancer cells than in HEK293T cells or gastric cancer cells (Fig. 1b). Furthermore, we found that ARHGEF16 protein levels were significantly increased in colon cancer tissue samples compared with paired adjacent normal tissue samples as detected by immunohistochemistry (IHC; Fig. 1c–e). These results suggested that ARHGEF16 is highly expressed in colon cancers.

We further analyzed the correlations between the expression of ARHGEF16 and the clinical and pathological features of patients with colon cancer. An immunoreactive score, which ranged from 0 to 12, was used to estimate the expression levels of ARHGEF16; we defined a score of <6 as low expression and a score 6 or more as high expression. The expression of ARHGEF16 in colon cancer samples was positively correlated with the degree of differentiation (*P* = 0.016; Table 1). However, the expression of ARHGEF16 in colon cancer samples had no correlations with sex, age, tumor size, lymphatic invasion, or TNM stage (Table 1). Taken together, these results demonstrated that ARHGEF16 could be a prognostic biomarker in human colon cancer.

**Table 1:** Association of ARHGEF16 expression levels with clinicopathologic characteristics in colon cancer.

Clinicopathologic characteristics	*n*	ARHGEF16 expression				*P* value
		Low (%)		High (%)		
Gender	71					
Male		6	8.5%	41	57.7%	0.871
Female		2	2.8%	22	31.0%	
Age (y)	71					
<60		3	4.2%	38	53.5%	0.395
≥60		5	7.0%	25	35.2%	
Tumor size (cm)	71					
<5		2	2.8%	28	39.4%	0.504
≥5		6	8.5%	35	49.3%	
Differentiation	71					
Well		4	5.6%	7	9.9%	0.016
Moderate		3	4.2%	34	47.9%	
Poor		1	1.4%	22	31.0%	
Lymph node metastasis	71					
Yes		3	4.2%	11	15.50%	0.384
No		5	7.0%	52	73.2%	
TNM stage	71					
I + II		5	7.0%	51	71.8%	0.456
III + IV		3	4.2%	12	16.9%	

### ARHGEF16 promotes the malignancy of colon cancer cells

**Fig. 2:**
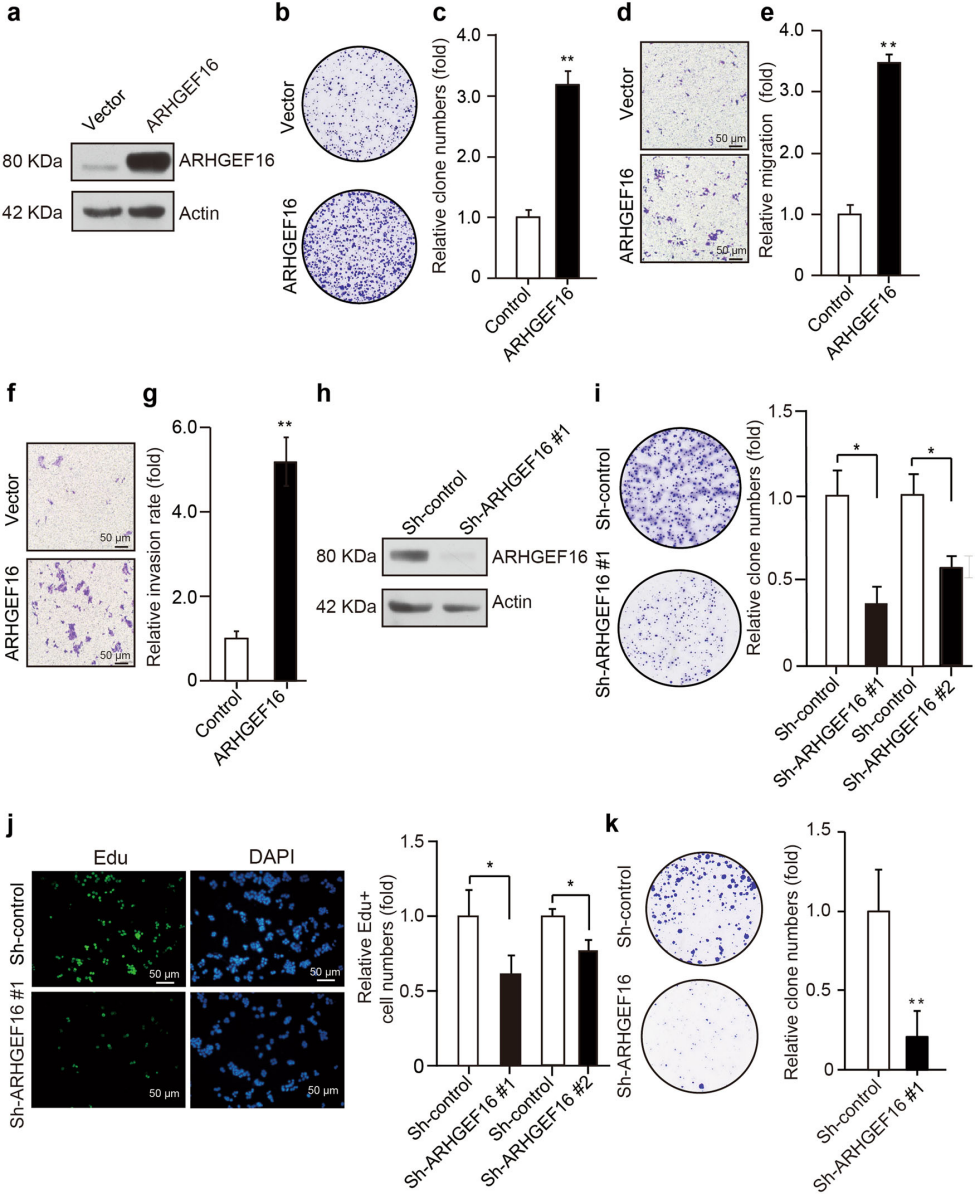
ARHGEF16 promotes colorectal cancer cell proliferation. **a** HCT116 cells were transfected with Vector or Lv-ARHGEF16 for 48 h and harvested for WB analysis with the indicated antibodies. **b** ARHGEF16 overexpression increased the colony formation ability of HCT116 cells. **c** Quantification of the colony formation rates was shown in Fig. 2b. Data are shown as the mean ± SD (*n* = 5). *P*-values were obtained by the two-side Student’s *t* test. **P < 0.01. **d** ARHGEF16 overexpression increased the migration of HCT116 cells. **e** Quantification of the migration rates was shown in Fig. 2d. Data are shown as the mean ± SD (*n* = 5). **P < 0.01. *P-*values were obtained by the two-side Student’s *t* test. **f** ARHGEF16 overexpression increased the invasion of HCT116 cells. **g** Quantification of the invasion rates was shown in Fig. 2f. Data are shown as the mean ± SD (*n* = 5). **P < 0.01. *P*-values were obtained by the two-side Student’s *t* test. **h** SW620 cells were transfected with Sh-control or Sh-ARHGEF16 #1 and harvested for WB analysis with the indicated antibodies. **i** Knockdown of ARHGEF16 decreased the colony formation ability of SW620 cells. SW620 cells were transfected with Sh-control or Sh-ARHGEF16 #1 and Sh-ARHGEF16 #2. *P*-values were obtained by the two-side Student’s *t* test. Data are shown as the mean ± SD (*n* = 5). *P < 0.05. **j** ARHGEF16 knockdown with Sh-control or Sh-ARHGEF16 #1 and Sh-ARHGEF16 #2 decreased the proliferation rate of SW620 cells, as shown by EdU staining. *P*-values were obtained by the two-side Student’s *t* test. Data are shown as the mean ± SD (*n* = 5). *P < 0.05. **k** ARHGEF16 knockdown with Sh-control or Sh-ARHGEF16 #1 decreased the colony formation ability of HT29 cells. **P < 0.01. *P*-values were obtained by the two-side Student’s *t* test. Data are shown as the mean ± SD (*n* = 5).

To test whether ARHGEF16 regulates the malignancy of colon cancer cells, we used a lentiviral system to stably express ARHGEF16 in HCT116 and SW480 cells that contain relatively low levels of this protein and to silence ARHGEF16 expression in SW620 and HT29 cells because these cells have relatively high endogenous levels of ARHGEF16. The overexpression or knockdown of ARHGEF16 in these cell lines was evaluated by western blot analysis (Fig. 2a, h and Supplementary Fig. S1a–c). Then, we found that overexpression of ARHGEF16 dramatically promoted the proliferation, migration, and invasion of the cells (Fig. 2b–g and Supplementary Fig. S1d–g). Knocking down ARHGEF16 expression dramatically inhibited the proliferation of the cells (Fig. 2i–k). Furthermore, we observed efficient knockdown of ARHGEF16 protein level by Sh-ARHGEF16 #1 than by Sh-ARHGEF16 #2 (Fig. 2h and Supplementary Fig. S1a). Therefore, we used Sh-ARHGEF16 #1 for targeting ARHGEF16 in further experiments. Exogenous ARHGEF16 could rescue the HCT116 cells proliferation inhibition, which was caused by ARHGEF16 knockdown (Supplementary Fig. S2a–c). These results demonstrated that ARHGEF16 played crucial roles in the proliferation and migration of colon cancer cells.

### ARHGEF16 accelerates colon carcinogenesis in vivo

**Fig. 3:**
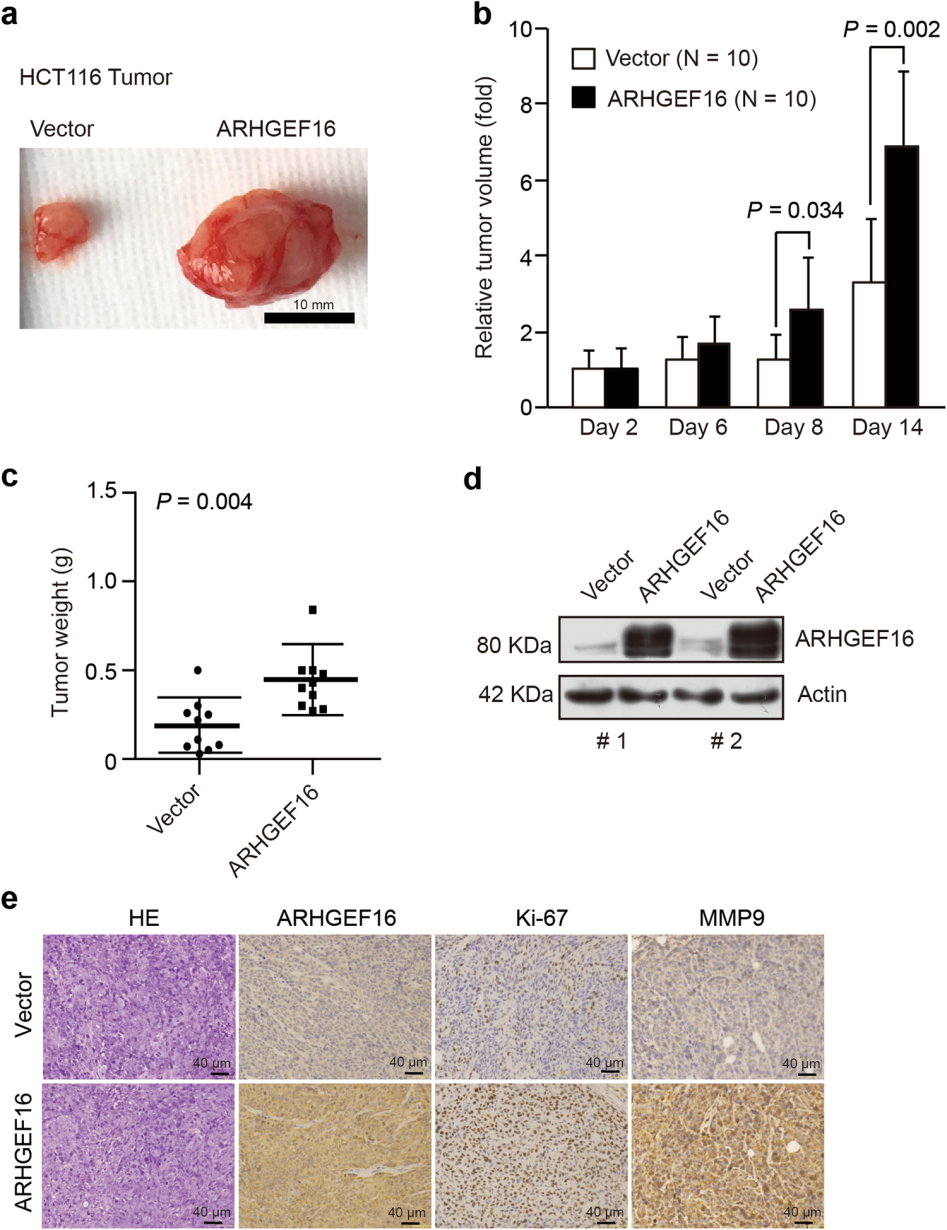
ARHGEF16 promotes colorectal carcinogenesis in vivo. **a** Overexpression of ARHGEF16 promoted tumor growth. Nude mice were injected subcutaneously with 2 × 10^7^ cells. Representative images of tumors developed in nude mice are shown. **b**, **c** Tumor growth in nude mice were injected subcutaneously with HCT116-Vector and HCT116-ARHGEF16 cell lines. *P*-values were obtained by the two-side Student’s *t* test. Data are shown as the mean ± SD (*n* = 10). **d** Overexpression of ARHGEF16 in xenografts was confirmed by WB analysis. **e** HE staining of tumor tissue samples from the indicated groups and detection of ARHGEF16, Ki67, and MMP9 protein levels by immunohistochemistry.

To demonstrate whether the above findings can be reproduced in vivo, we generated a colon cancer tumor xenograft mouse model. HCT116 or SW480 cell lines ectopically expressing ARHGEF16 were subcutaneously injected into the flanks of nude mice. Then, the expression of ARHGEF16 in the xenograft tumors was determined by western blotting (Fig. 3d and Supplementary Fig. S1j).The overexpression of ARHGEF16 led to dramatic increases in the average tumor volume (by ∼6-fold; Fig. 3a, b) and the average tumor weight (by ∼3-fold; Fig. 3c and Supplementary Fig. S1h, i) compared with control expression. IHC results indicated that the expression of the proliferation marker Ki67 and that of MMP9, which is involved in cancer invasion and metastasis, was significantly increased in the ARHGEF16-overexpressing xenograft tumors (Fig. 3e). These results suggest that ARHGEF16 plays a key role in driving the growth of xenograft colon tumors.

### FYN is identified as an ARHGEF16-interacting partner

**Fig. 4:**
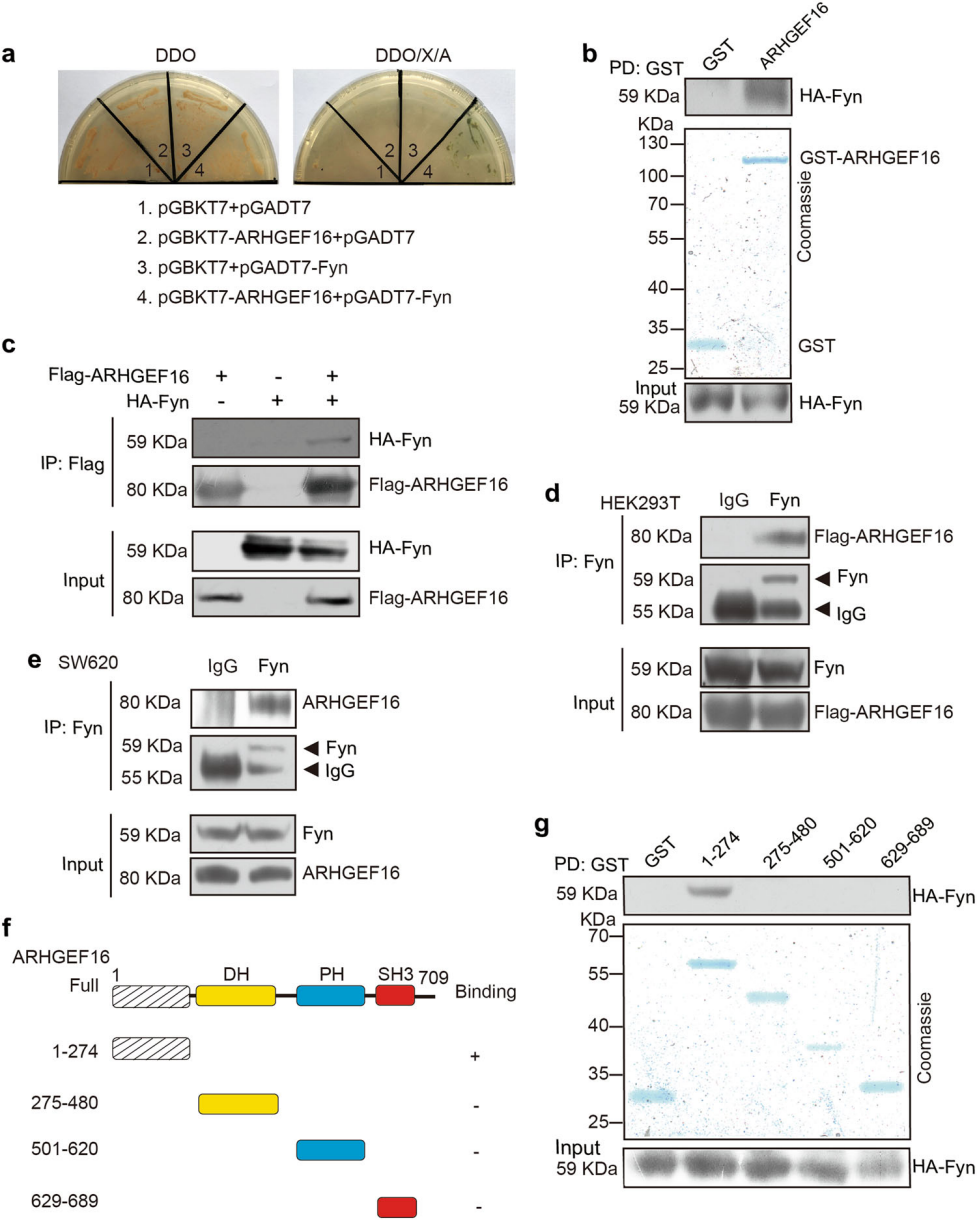
ARHGEF16 interacts directly with a FYN. **a** The interaction of ARHGEF16 with FYN was analyzed by a yeast two-hybrid screen. **b** ARHGEF16 interacted with FYN. Bacteria-expressed GST-ARHGEF16 immobilized on Glutathione Sepharose 4B beads was incubated with lysates from HEK293T cells transfected with HA-FYN. The precipitated proteins and input lysates were subjected to SDS–PAGE and WB analysis. **c** Interaction of ARHGEF16 with FYN in mammalian cells. HEK293T cell lysates cotransfected with Flag-ARHGEF16 and HA-FYN or an empty vector were incubated with an anti-Flag antibody. The immunoprecipitate (IP) and input lysates were probed with the indicated antibodies. **d** Flag-ARHGEF16 bound to endogenous FYN in HEK293T cells. HEK293T cell extracts were subjected to IP with beads coated with normal rabbit IgG or the anti-FYN antibody. The resulting precipitates were probed with the indicated antibodies. **e** Endogenous ARHGEF16 bound to FYN in SW620 cells. SW620 cell extracts were subjected to IP with beads coated with normal rabbit IgG or the anti-FYN antibody. **f** Schematic illustration of the domains of ARHGEF16. **g** Mapping the domains responsible for the ARHGEF16-FYN interaction. Bacteria-expressed GST-Full or truncated ARHGEF16 immobilized on Glutathione Sepharose 4B beads was incubated with lysates from HEK293T cells transfected with HA-FYN. The precipitated proteins and input lysates were subjected to SDS–PAGE and WB analysis.

Although it is well established that ARHGEF16 plays a central role in tumorigenesis, the potential mechanism(s) underlying its function remains poorly understood. To address this issue, we searched for ARHGEF16-interacting proteins by performing a yeast two-hybrid screen using human ARHGEF16 as the bait and isolated FYN as a potential ARHGEF16-interacting protein (Fig. 4a). To verify this finding, we performed an in vitro GST pull-down assay using HA-FYN expressed in HEK293T cells and purified GST-ARHGEF16. As shown in Fig. 4b, recombinant HA-FYN was pulled down with GST-ARHGEF16. This ARHGEF16-FYN interaction was direct, as confirmed by another coimmunoprecipitation (IP) experiment (Fig. 4c). Consistent with these results, a Flag-ARHGEF16 protein could be immunoprecipitated with an anti-FYN antibody (Fig. 4d), indicating that ARHGEF16 and FYN can form a protein complex in HEK293T cells. We also detected the endogenous ARHGEF16-FYN complex by co-IP analysis in SW620 cells (Fig. 4e). Next, we tried to map the binding domains of these proteins by using different fragments of these two proteins and constructing several truncated ARHGEF16 proteins (Fig. 4f). An in vitro GST-fusion protein pull-down assay validated the direct binding of a domain (1–274) of ARHGEF16 to FYN (Fig. 4g).

Taken together, these results identified FYN as a novel ARHGEF16-binding protein and demonstrated that the N-terminus of ARHGEF16 directly interacted with FYN.

### FYN-ARHGEF16 axis promotes colon cancer cell proliferation and migration

**Fig. 5:**
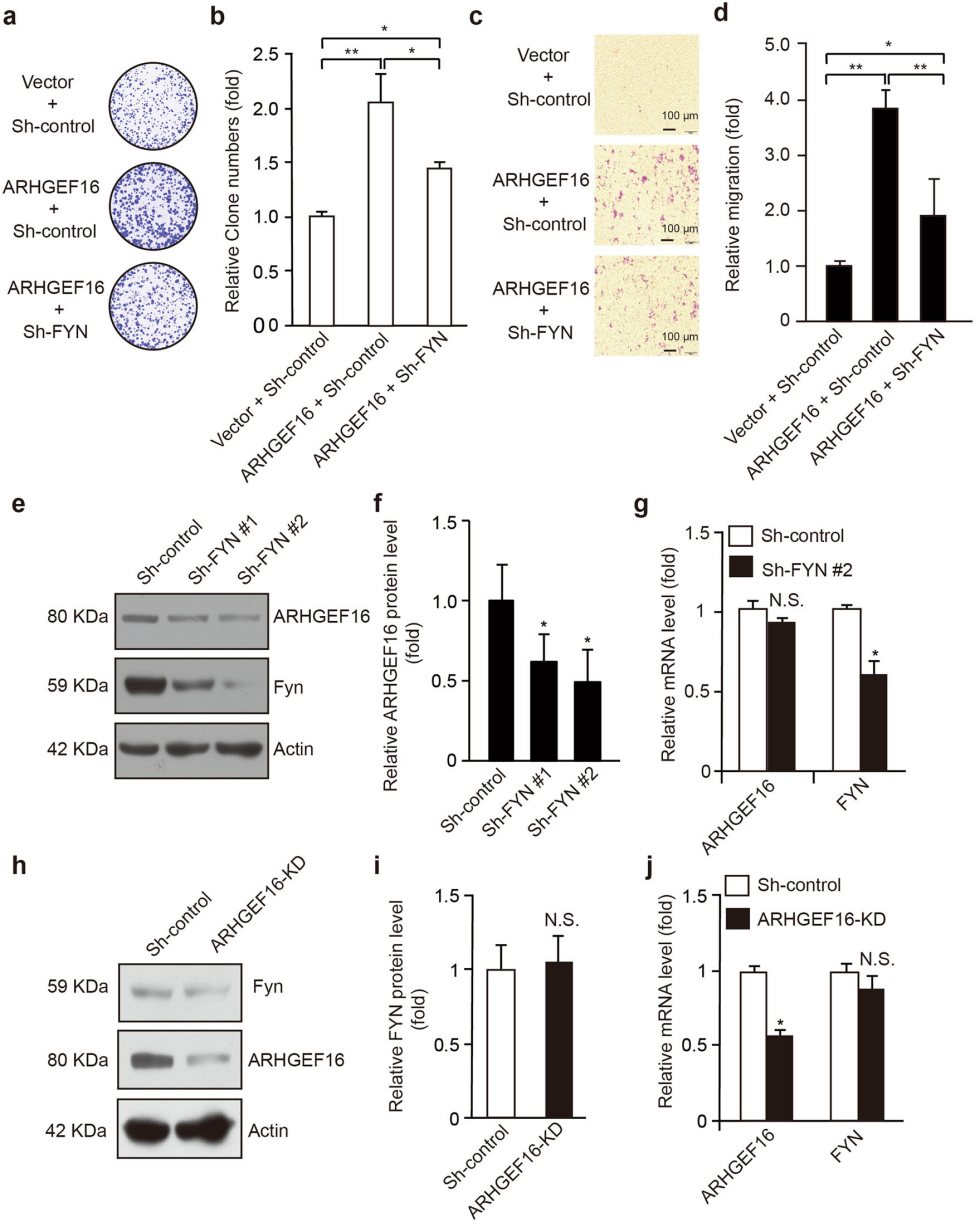
Knocking down FYN expression inhibits the ARHGEF16-mediated promotion of cell migration and proliferation. **a** Knocking down FYN expression repressed the ARHGEF16-induced proliferation of colon cancer cells. The proliferation ability of HCT116 cells stably expressing Vector + Sh-control, ARHGEF16 + Sh-control and ARHGEF16 + Sh-FYN were examined using a colony formation assay. **b** Quantification of cell migration rates in Fig. 5a. *P*-values were obtained by the two-side Student’s *t* test. Data are shown as the mean ± SD (*n* = 5). *P < 0.05. **c**, **d** Knocking down FYN expression decreased the ARHGEF16-stimulated migration of HCT116 cells, and representative images of filters stained with crystal violet are shown. **P < 0.01. *P-*values were obtained by the two-side Student’s *t* test. Data are shown as the mean ± SD (*n* = 5). **e-g** Knocking down FYN expression decreased the ARHGEF16 protein level but not the mRNA level. The results of the WB analysis are presented in **e**, Protein level **f**, and mRNA level **g**. *P*-values were obtained by the two-side Student’s *t* test. Data are shown as the mean ± SD (*n* = 3). *P < 0.05. **h-j** Knocking down ARHGEF16 expression did not affect the FYN protein level or mRNA level. The results of a Western blotting analysis are presented in **h**. Protein level **i**, and mRNA level **j**. *P*-values were obtained by the two-side Student’s *t* test. Data are shown as the mean ± SD (*n* = 3). N.S. not significant.

Based on the above manifestations, we assumed that FYN was an effector in the context of ARHGEF16-induced colon progression. To verify this hypothesis, we knocked down FYN expression in HCT116 cells that stably expressed ARHGEF16 using a lentiviral system and confirmed the knockdown by WB analysis (Supplementary Fig. S3a). We first analyzed cell proliferation by assessing colony formation and found that it was increased in ARHGEF16 + Sh-control cells relative to Vector + Sh-control cells, but this increase was abrogated by knocking down FYN expression (ARHGEF16 + Sh-FYN; Fig. 5a, b), suggesting that ARHGEF16-induced colon cancer cell proliferation was regulated by FYN. Moreover, cell migration was also inhibited in ARHGEF16 + Sh-FYN HCT116 cells relative to ARHGEF16 + Sh-control HCT116 cells (Fig. 5c, d), suggesting that FYN is required for ARHGEF16-induced colon cancer cell migration. Consistent with the above results, knocking down FYN expression resulted in a decrease in the ARHGEF16 protein level (Fig. 5e, f) but not the mRNA level (Fig. 5g). In contrast, knocking down ARHGEF16 expression did not affect the protein or mRNA level of FYN (Fig. 5h–j).

**Fig. 6:**
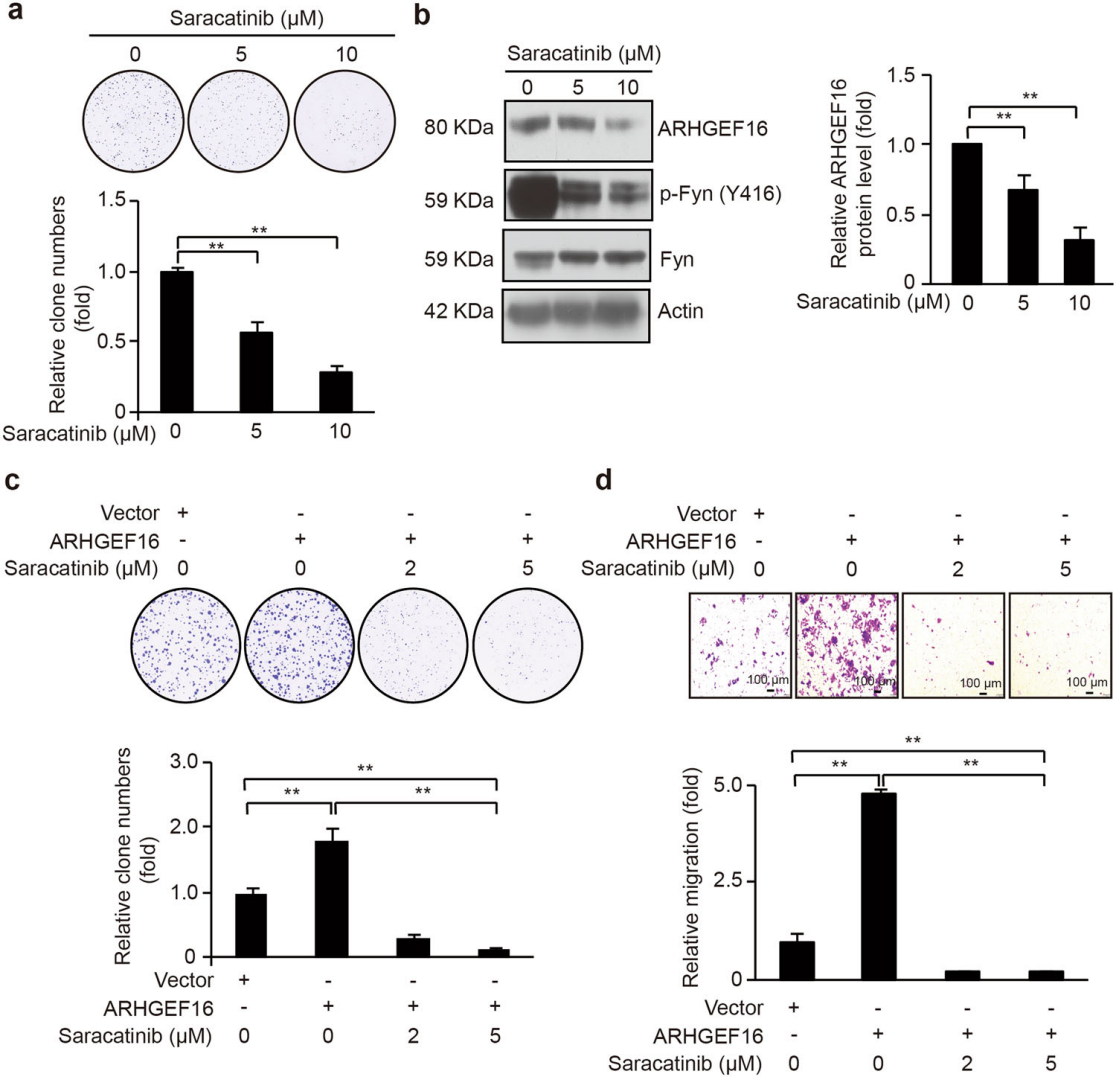
Saracatinib targeting FYN reduced ARHGEF16 protein level, and inhibited ARHGEF16-mediated colon cancer cell proliferation and migration. **a** The effect of Saracatinib on the colony formation ability of SW620 cells. Data are shown as the mean ± SD (*n* = 5). **P < 0.01. *P*-values were obtained by the two-side Student’s *t* test. **b** Saracatinib decreased the ARHGEF16 protein level in SW620 cells. SW620 cells were treated with Saracatinib for 24 h. The relative intensity value was calculated with the NIH ImageJ software using basal level of β-actin as an internal control. Data are shown as the mean ± SD (*n* = 3). **P < 0.01. *P-*values were obtained by the two-side Student’s *t* test. **c** Saracatinib *t*reatment repressed the ARHGEF16-induced proliferation of colon cancer cells. The proliferation ability of HCT116 cells stably expressing Vector and ARHGEF16 were examined using a colony formation assay. *P*-values were obtained by the two-side Student’s *t* test. Data are shown as the mean ± SD (*n* = 5). **P < 0.01. **d** Saracatinib treatment decreased the ARHGEF16-stimulated migration of HCT116 cells, and representative images of filters stained with crystal violet are shown. **P < 0.01. *P*-values were obtained by the two-side Student’s *t* test. Data are shown as the mean ± SD (*n* = 5).

Saracatinib (AZD0530) is an anilinoquinazoline that has been widely used as an inhibitor for Src. Saracatinib treatment suppresses the metastasis of bladder cancer in a murine model and prostate cancer cell proliferation^18,19^. Saracatinib treatment could also reduce the phosphorylation of Fyn (Y416) in hepatic stellate cells^20^. To examine the effect of saracatinib on the ability of ARHGEF16-induced colon cancer cell proliferation, we first performed colony formation assays. We observed that Saracatinib treatment significantly decreased SW620 cell proliferation (Fig. 6a). Next, we analyzed the effect of Saracatinib treatment on ARHGEF16 protein level in the cells. Western blot analysis, we detected that the degree of reduced ARHGEF16 level was in proportional to the decreased level of p-Y416-Fyn by Saracatinib treatment (Fig. 6b). Furthermore, we found that Saracatinib treatment inhibited ARHGEF16-overexpressing HCT116 cell proliferation and migration compared with the cells in the absence of Saracatinib, indicating the inhibitory role of Saracatinib for ARHGEF16-induced colon cancer cell proliferation and migration (Fig. 6c, d), in line with the results of FYN knockdown.

Taken together, these results indicate that the FYN-ARHGEF16 axis plays crucial roles in promoting colon cancer cell proliferation and migration.

### FYN increases the stability of ARHGEF16 by inhibiting ARHGEF16 degradation

**Fig. 7:**
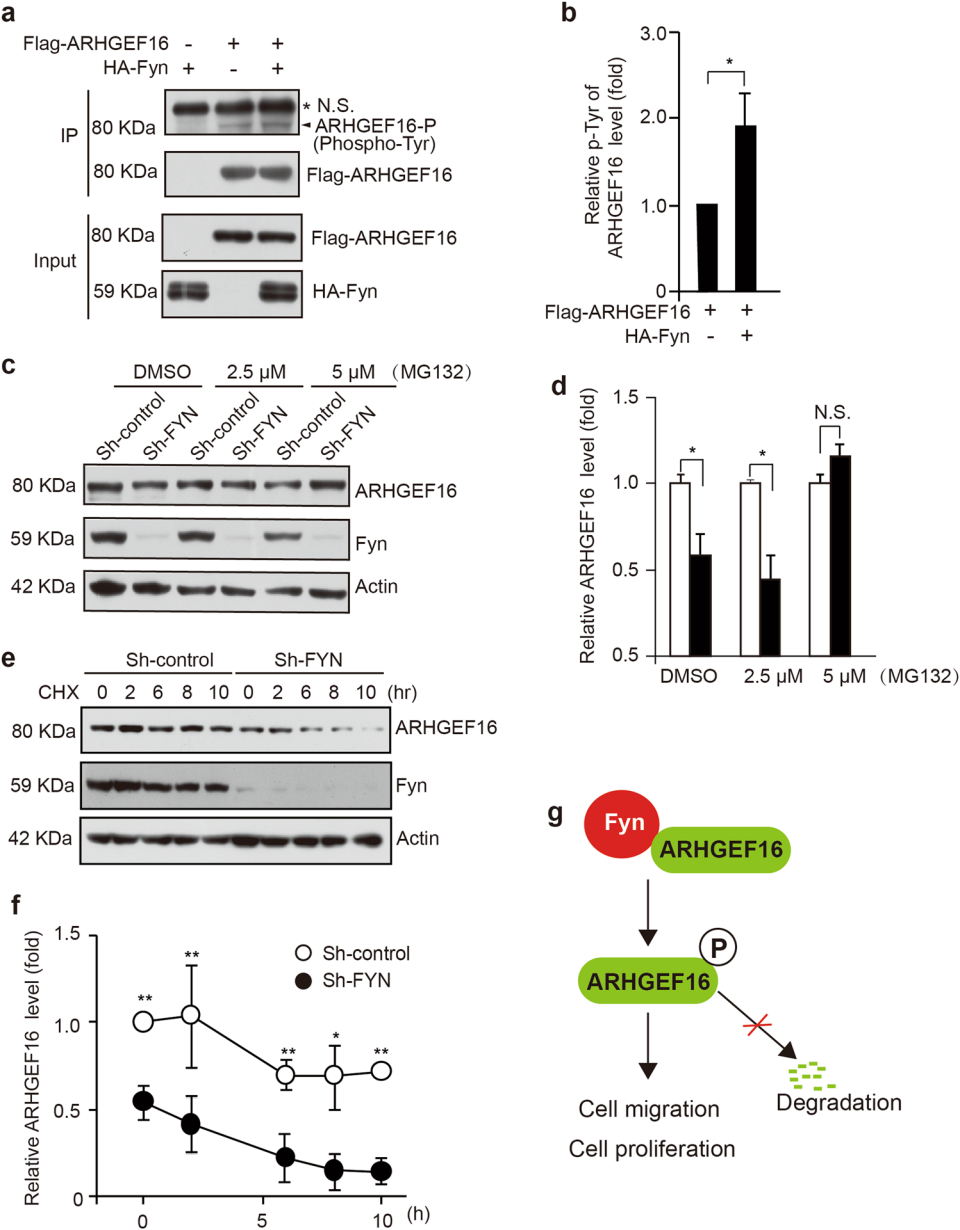
ARHGEF16 is stabilized by FYN. **a**, **b** Cell lysates from HEK293T cells transfected with the indicated plasmids were immunoprecipitated with an anti-Flag antibody and total cell lysates were analyzed with antibodies against Flag, HA and phosphor-Tyr. The relative intensity value was calculated with the NIH ImageJ software using basal level of p-Tyr as an internal control. *P*-values were obtained by the two-side Student’s *t* test. Data are shown as the mean ± SD (*n* = 3). *P < 0.05. N.S. not significant. **c**, **d** Knocking down FYN expression stimulated the proteolysis of ARHGEF16 by the proteasome. SW620 Sh-Control or Sh-FYN cells were treated with the proteasome inhibitor MG132 for 8 h, and the cells were subsequently subjected to immunoblotting with the indicated antibodies. DMSO, dimethylsulfoxide. The relative intensity value was calculated with the NIH ImageJ software using β-actin as an internal control. *P*-values were obtained by the two-side Student’s *t* test. Data are shown as the mean ± SD (*n* = 3). *P < 0.05. N.S. not significant. **e, f** Knocking down FYN expression reduced the half-life of endogenous ARHGEF16. SW620 Sh-Control or Sh-FYN cells were treated with the protein synthesis inhibitor CHX (10 μg/ml) for the indicated times, and the cells were subsequently subjected to immunoblotting with the indicated antibodies. The relative intensity value was calculated with the NIH ImageJ software using β-actin as an internal control. *P*-values were obtained by the two-side Student’s *t* test. Data are shown as the mean ± SD (*n* = 3). *P < 0.05. **g** Model for FYN-ARHGEF16 axis-mediated colon cancer cell proliferation. The suggested molecular circuitry controlling the proliferation and migration in colon cancer cells, based on the ARHGEF16 and FYN proteins. Knocking down FYN expression promoted the degradation of ARHGEF16, which in turn led to reduced proliferation and migration in colon cancer cells.

To elucidate the possible role of FYN in regulating the ARHGEF16 protein level, we performed several experiments to investigate the role of FYN in ARHGEF16 stabilization. We first examined the effects of different levels of FYN expression on ARHGEF16 phosphorylation in HEK293T cells. As shown in Fig. 7a, the phosphorylation level of Flag-ARHGEF16 was increased concomitantly upon enhanced HA-FYN expression, indicating that ARHGEF16 was tyrosine phosphorylated in the presence of FYN (Fig. 7b). Furthermore, we observed that the phosphorylation level of Flag-ARHGEF16 was partially reduced by FYN knockdown in SW620 cells (Supplementary Fig. S3b). To investigate whether the degradation of ARHGEF16 is regulated by a proteasome-dependent mechanism, SW620 cells with knocked down FYN expression were treated with MG132 (a proteasome inhibitor) for 8 h to inhibit proteasome activity. Western blot results showed that MG132 treatment increased ARHGEF16 expression at the protein level in a dose-dependent manner (Fig. 7c, d), suggesting that FYN stabilizes ARHGEF16 in a proteasome-dependent manner. Furthermore, the half-life of ARHGEF16 was evaluated in the presence of cycloheximide (CHX) since CHX can inhibit protein synthesis. As shown in Fig. 7e, knocking down FYN expression in SW620 cells significantly shortened the half-life of endogenous ARHGEF16 at 8 h and 10 h (Fig. 7f). Knocking down FYN expression resulted in facilitated degradation of exogenous ARHGEF16, suggesting that ARHGEF16 is specifically stabilized by FYN. These results indicate that the FYN–ARHGEF16 axis promotes colon cancer progression and that knocking down FYN expression increases proteasome-dependent ARHGEF16 degradation (Fig. 7g). However, which tyrosine residue(s) of ARHGF16 is phosphorylated by FYN could not be identified in this study. We will approach this matter in future work.

## Discussion

ARHGEF16 is a GEF in the Rho GTPase family, whose members regulate cell morphogenesis, proliferation, invasion, and survival through regulation of the actin cytoskeleton^2–6,11^. ARHGEF16, also known as Ephexin 4, can bind to the cytoplasmic region of the Ephrin receptor. Ephrin signaling plays a key role in cellular repulsion, attraction, and migration by controlling local cytoskeletal dynamics through Ephexin proteins and Rho GTPases^8,12^. Dysregulation of ARHGEF16 contributes to carcinogenesis and tumor progression^13,21^. However, how ARHGEF16 is regulated in response to the progression of colon cancer remains poorly understood. In this study, we investigated the effects of ARHGEF16 on the progression of colon cancer cells in vitro and in vivo and found that overexpression of ARHGEF16 caused marked increases in the proliferation and migration of colon cancer cells in vitro and in vivo. In contrast, knocking down ARHGEF16 expression reduced the proliferation, migration, and invasion of colon cancer cells. We also found that ARHGEF16 levels were highly correlated with tumor differentiation in colon cancers by analyzing clinical colon cancer specimens. Our findings highlight the importance of ARHGEF16 in the regulation of colon cancer progression.

The present study shows that ARHGEF16 is a key factor in maintaining tumorigenesis, but it remains unknown whether other specific factors regulate the activity of ARHGEF16. In this study, we identified FYN as a novel partner of ARHGEF16 through assays evaluating direct binding to ARHGEF16. We further validated that ARHGEF16 promoted proliferation and migration in colon cancer cells, which were strongly dependent on FYN. Thus, this is a novel mechanism that regulates ARHGEF16 activity involved in promoting the progression of colon cancer.

FYN is a nonreceptor tyrosine kinase in the Src family of kinases that plays critical roles in the development and progression of many cancers by regulating morphogenic transformation, cellular motility, cell growth, and cell death^16,17^. Furthermore, activation of PIKE-A by FYN is important for the oncogenic activities of AMPK signaling because the tumor suppressive function of AMPK is impaired^22^. Interestingly, FYN has been shown to be an important effector in the HGF/MET signaling axis that acts as a crucial oncoprotein in prostate cancer metastasis^23^. FYN is also highly expressed in tamoxifen-resistant estrogen receptor-positive (ER^+^) breast cancer cell lines mainly because it plays an important role in the activation of important cell-cycle-associated proteins such as 14-3-3 and Cdc25A, ultimately contributing to overcoming the antiproliferative effects of tamoxifen^17,24^. FYN is highly associated with the growth and migration of glioma cell lines^25,26^. Furthermore, recent evidence has shown that FYN plays a critical role in the metastatic ability of basal-type breast cancer cells via the STAT5/NOTCH2 signaling axis^27^. Thus, elevated expression and/or activation of FYN is observed in various cancers including glioblastoma, melanoma, squamous cell carcinoma, prostate, and breast cancers, and FYN is an oncoprotein important for cancer cell proliferation and growth^16,17,24,25,27,28^. Therefore, FYN is considered a pivotal oncogene as a result of its regulatory roles in cancer-related signaling pathways. Interestingly, we found that overexpression of FYN increased the tyrosine phosphorylation level of ARHGEF16. FYN is also required for stabilization of ARHGEF16. Moreover, we found that FYN was required for the ARHGEF16-mediated promotion of proliferation, which might be mediated by FYN altering cellular ARHGEF16 protein stability. Thus, our study provides credible evidence via a series of independent experiments that FYN interacts with ARHGEF16, a critical regulator of Ephrin signaling, and FYN kinase activity may be responsible for ARHGEF16 phosphorylation and stabilization.

The Ephexin subfamily comprises five members: Ephexin1 (ARHGEF27/ NGEF), Ephexin2 (ARHGEF19/WGEF), Ephexin3 (ARHGEF5/TIM1), Ephexin4 (ARHGEF16), and Ephexin5 (ARHGEF15/Vsm-RhoGEF)^9,21,29–32^. It has been reported that Ephexin1 and Ephexin5 are phosphorylated by Src or Eph receptors but not ARHGEF16^21,29,33^. Here, we showed that FYN stabilized ARHGEF16 by promoting ARHGEF16 phosphorylation in a manner dependent on FYN. Thus, the mechanisms regulating Ephexin activity by tyrosine phosphorylation are different from one another. Our findings may represent in part how ARHGEF16 is persistently activated in colon cancer cells. However, it is unknown whether ARHGEF16 exerts its function in a manner dependent on FYN-mediated phosphorylation, and this issue remains to be clarified.

In summary, our findings suggest that ARHGEF16 contributes to the proliferative ability of colon cancer cells through FYN. Thus, co-targeting ARHGEF16 and FYN maybe a relatively effective approach for anti-colon cancer therapy, as these molecules are highly active in and related to colon cancer. Although additional studies are needed to understand whether the FYN-ARHGEF16 signaling axis promotes colon cancer progression through some other mechanisms and whether this axis works in other tumors, our current study offers useful information for future precision oncology with a new biomarker for this type of cancer.

## Materials and methods

### Plasmid construction, reagents, and antibodies

A silencing construct was constructed with a BLOCK-iT Pol II miR RNAi Expression Vector kit (Invitrogen, Carlsbad, CA, USA; K4936–00); lentiviruses (LVs) for overexpressing ARHGEF16 or knocking down ARHGEF16 expression were obtained from GeneChem (Shanghai, China). The GV358 and GV307 LV vectors were used for overexpression and knockdown, respectively. Human FYN overexpression and silencing were mediated by phage and pSUPER RNAi systems, respectively. The target sequences are shown in Additional file 1: Table S1. FYN was subcloned into the region between the NotI and EcoRI sites of pKH3 and the EcoRI and BamHI sites of pGADT7. Human full-length ARHGEF16 was subcloned into the region between the EcoRI and BamHI sites of pGEX-6P-1 and the EcoRI and BamHI sites of pGBKT7. Fragments of ARHGEF16 (residues 1–274, 275-480, 501–620, and 629–689) were inserted into the region between the EcoRI and BamHI sites of pGEX-6P-1.

A protease inhibitor cocktail was purchased from Sigma-Aldrich (St. Louis, MO, USA). Puromycin was purchased from GeneChem (Shanghai, China) or Solarbio (Beijing, China). TRIzol reagent (#15596018) and Lipofectamine 2000 transfection reagent (#11668019) were purchased from Thermo Fisher Scientific (Waltham, MA, USA). Protein G agarose beads (#11243233001) and Protein A agarose beads (#11134515001) were purchased from Roche (Palo Alto, CA, USA), and Glutathione Sepharose 4B beads (#17–0756-01) were purchased from GE Healthcare (Little Chalfont, UK). Saracatinib was purchased from MCE (Monmouth Junction, NJ, USA). Antibodies against the following proteins were used for western blotting: phospho-Tyr mouse mAb (#9416), ARHGEF16 (ab86068), β-actin (Santa Cruz Biotechnology, Santa Cruz, CA, USA; sc-1616-R), Flag (F3165), FYN (#4023), and phospho-Y416-Fyn (#6943).

### Yeast two-hybrid screening

Yeast two-hybrid screening was performed with the Matchmaker Gold Yeast Two-Hybrid System and Universal Human Mate &Plate™ Library (Clontech Laboratories). The ARHGEF16 gene was cloned into the pGBKT7 vector as the bait and subsequently transformed into the *Saccharomyces cerevisiae* Y2HGold strain growing on SD/-Trp medium according to the company’s protocol. The Y187 yeast strain containing the cDNA library was mated with Y2HGold yeast containing the ARHGEF16 expression vector. Positive blue colonies growing on SD/-Leu/-Trp/X-α-Gal/Aba (DDO/X/A) medium were selected. The positive cDNA clones were amplified by PCR using the T7 sequencing primer, followed by sequencing to identify genes.

### Cell culture and transfection

The human colon cancer cell lines HCT116, SW480, HT29, and SW620 and the transformed human embryonic kidney cell line HEK293T were purchased from the American Type Culture Collection (ATCC; Manassas, VA). For transfection, cells were grown on coverslips in 35-mm-diameter culture dishes to ~70–80% confluence and transfected with the indicated plasmids utilizing Lipofectamine 3000 (Invitrogen) according to the manufacturer’s instructions. Cells were cultured at 37 °C in an atmosphere of 5% CO_2_ and 95% humidity.

### RNA extraction and RNA interference

Total RNA was extracted from cells by TRIzol^®^ Reagent (#15596018) and evaluated by real-time PCR. Briefly, 1 μg of total RNA was employed to generate cDNA via reverse transcription using the PrimeScript^®^ RT reagent Kit containing gDNA Eraser (Takara, DRR047A). Real-time PCR was performed using SYBR^®^Premix Ex Taq™TliRnaseH Plus (Takara, DRR820A) with the ABI StepOnePlus™ Real-Time PCR System (Applied Biosystems, Foster, City, CA). GAPDH, as an internal control, was employed to standardize any discrepancies in expression levels. The sequences of the oligonucleotide specific for FYN or ARHGEF16 are listed in file 1: Table S2. Cell transfection was implemented according to the protocol provided in the manufacturer’s instructions.

### Immunoprecipitation and western blot analysis

To detect the interaction between ARHGEF16 and FYN, a cell lysate was incubated with Flag beads in a lysis buffer (25 mM Tris–HCl, pH 7.0; 1 mM EDTA; 300 mM NaCl; 10% glycerol; 1% NP-40; 1 mM DTT; 10 mM NaF; 25 mM DMSF; and an EDTA-free protease inhibitor tablet (Complete: Roche)) overnight at 4 °C. After washing with the lysis buffer, the beads were denatured at 95 °C in 1x sample buffer and evaluated by SDS–PAGE followed by immunoblotting.

### Immunohistochemistry

Paraffin sections (3 μm thick) of formalin-fixed colon cancer and adjacent tissue samples were evaluated. Tissue sections were dewaxed, rehydrated, and incubated in 10 mM sodium citrate buffer (pH 6.0) for 10 min and incubated with 10% normal goat serum to block nonspecific staining. The sections were exposed to the indicated antibodies at 4 °C in a humidified chamber overnight, and immunoreactivity was visualized using the Polink-2 HRP DAB Detection Kit according to the manufacturer’s procedure. Images were captured with an FSX100 microscope equipped with a digital camera system (Olympus). Samples were examined by at least two individual researchers to independently determine the histopathological features of the samples using the German semiquantitative scoring method. Each specimen was scored for the staining intensity of nuclear, cytoplasmic, or membrane staining (no staining = 0; weak staining = 1; moderate staining = 2; and strong staining = 3) and for the extent of cell staining (0% = 0, 1–24% = 1, 25–49% = 2, 50–74% = 3, and 75–100% = 4). The intensity score multiplied by the extent score was used to determine the final immunoreactive score, which ranged from 0 to 12, to indicate the expression of ARHGEF16. Sections were also stained by H&E to distinguish between colon cancer tissue and adjacent normal tissue.

### GST pull-down assay

To detect the interaction between ARHGEF16 or truncated ARHGEF16 and FYN, GST-fusion proteins were produced in BL21, purified, and immobilized on Glutathione Sepharose 4B beads (Amersham Pharmacia). The beads were then incubated with lysates from HEK293T cells transfected with HA-FYN. Bead-associated proteins were subjected to SDS–PAGE and WB analysis.

### Cell proliferation, migration, and invasion assays

Cell proliferation was evaluated with an EdU incorporation assay, which was performed using a Cell-Light EdU imaging detecting kit according to the manufacturer’s protocol (Ruibo Biotechnology, Guangzhou).

For a colony formation assay, approximately equal numbers of HCT116, SW480, HT29, and SW620 cells (∼3 × 10³/well) were seeded into six-well plates (with triplicate wells for each cell type) in DMEM supplemented with 10% fetal bovine serum (Gibco). After 2 weeks, the cells were stained with crystal violet. The positive colonies composed of more than 50 cells were counted under a microscope and evaluated by ImageJ software (National Institutes of Health, Bethesda, MD, USA).

Cell migration was evaluated using Transwell plates (8 μm pore size, 6.5 mm diameter; Corning Life Sciences, Lowell, MA). Briefly, 1 × 10^5^ cells in 100 μl of DMEM containing 1% FBS were plated in the upper part of the chambers. Then, 600 μl of DMEM supplemented with 10% FBS was added into the bottom wells. After culturing for 36 h, the lower surface of the membranes in the wells containing the cells was fixed using 4% paraformaldehyde and subsequently stained with crystal violet. The cell number was counted under an optical microscope. Each of these experiments was repeated at least three times.

Cell invasion assays were carried out with Transwell plates precoated with Matrigel Basement Membrane Matrix (1 mg/ml; BD Biosciences, Franklin Lakes, NJ) according to the manufacturer’s instructions. Briefly, 1 × 10^5^ cells in 200 μl of FBS-free medium were seeded in the upper part of the chambers. The bottom wells in the system were filled with 600 μl of DMEM supplemented with 10% FBS. After being cultured for 36 h, the cells on the lower surface of the membranes were fixed using 4% paraformaldehyde and subsequently stained with crystal violet. Cell numbers were determined under an optical microscope. Each of these experiments was repeated at least three times.

### In vivo xenograft experiment

HCT116 cells (2 × 10^7^cells) and SW480 cells (1 × 10^7^ cells) stably infected (Lv-Vector and Lv-ARHGEF16) were resuspended in sterile PBS (200 µl) and injected subcutaneously into both flanks of 5-week-old female BALB/c-nu mice (SLAC Laboratory Animal CO. Ltd, Hunan, China). Tumor sizes in both flanks of the mice were monitored by Vernier caliper thrice weekly. The nude mice were humanly treated under the guidelines of the animal care and use committee of the First Affiliated Hospital of Nanchang University, and conformed to the guidelines for the Care and Use of Laboratory Animals published by the National Institutes of Health.

### Human tissue specimens

Human specimens were retrieved via surgical intervention without prior radiotherapy or chemotherapy. All samples were collected at the First Affiliated Hospital of Nanchang University between 01/2009 and 08/2014, along with complete clinical data. The study protocol was approved by the Institutional Review Board of the First Affiliated Hospital of Nanchang University. All patients and control subjects provided written informed consent. We confirmed pathological diagnoses according to established WHO criteria, and TNM staging was determined according to the criteria of the UICC. Detailed clinical and pathological patient information is summarized in Table 1.

### Statistical analysis

Unless otherwise indicated, data are expressed as the mean ± SD of experiments performed at least three times. Differences between two groups were assessed with paired two-side Student’s *t* test, the *χ*^2^ test for linear-by linear association or Mann–Whitney *U* test. The sample size and power were calculated by G Power 3.1. Differences were considered significant if *P* < 0.05. All analyses were carried out using SPSS v.13.0 software (SPSS Inc., Chicago, IL).

## Supplementary information

Supplementary Figure S1

Supplementary Figure S2

Supplementary Figure S3

Supplementary Figure Legends

Stat. table for Figures

Supplementary Tables

HCT116 tumors

## Data Availability

The source data underlying Figs. 1–7 and Supplementary Figs. S1–3 are provided as a Source data file.
