# Supplementary figures and images for: FYN is required for ARHGEF16 to promote proliferation and migration in colon cancer cells
Source: Cell Death Dis. 2020 Aug 7;11(8):652. doi: 10.1038/s41419-020-02830-1 (PMC7435200; doi:10.1038/s41419-020-02830-1)

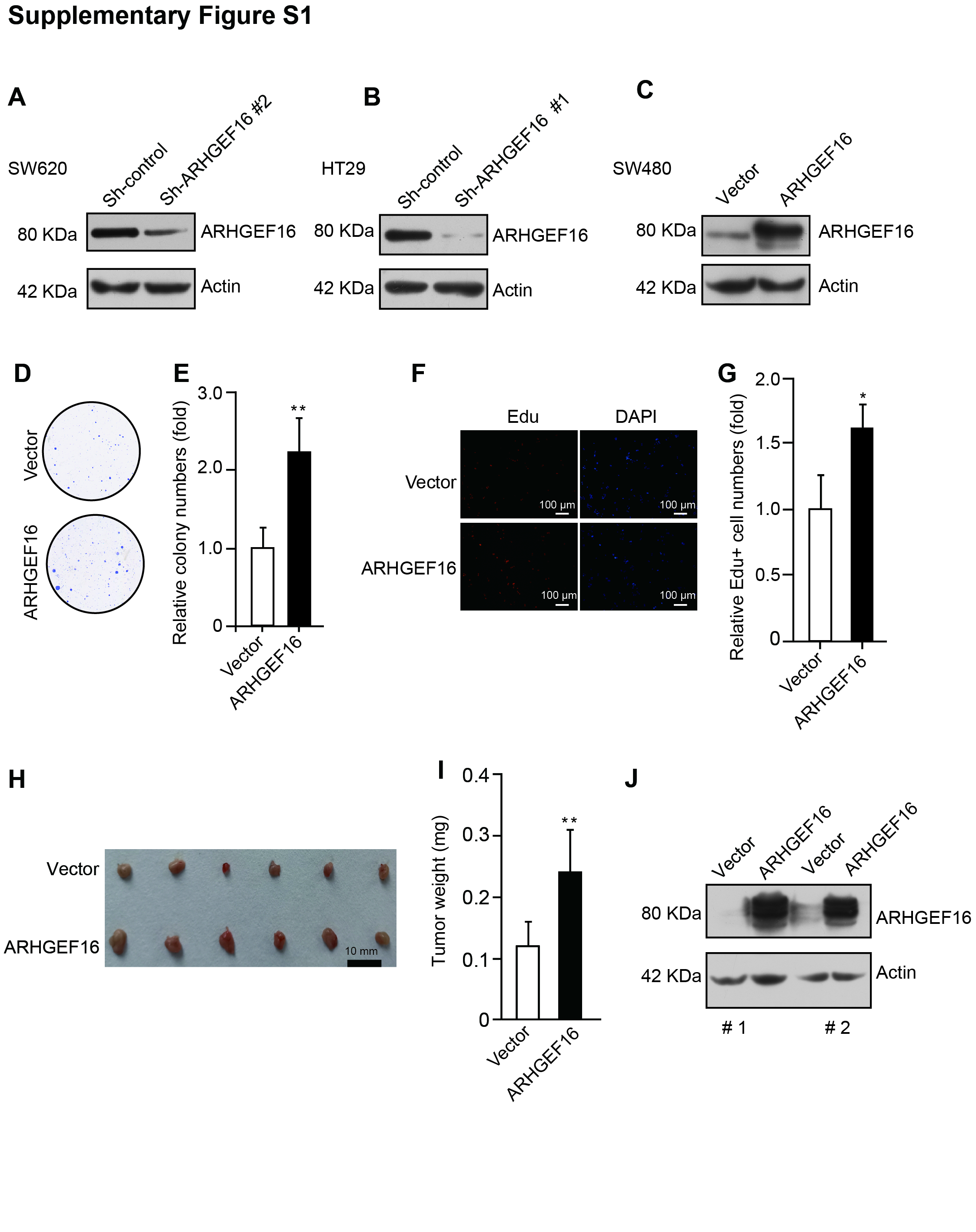

Supplement: Supplementary file 1 — Supplementary Figure S1 [file 41419_2020_2830_MOESM1_ESM.tif]

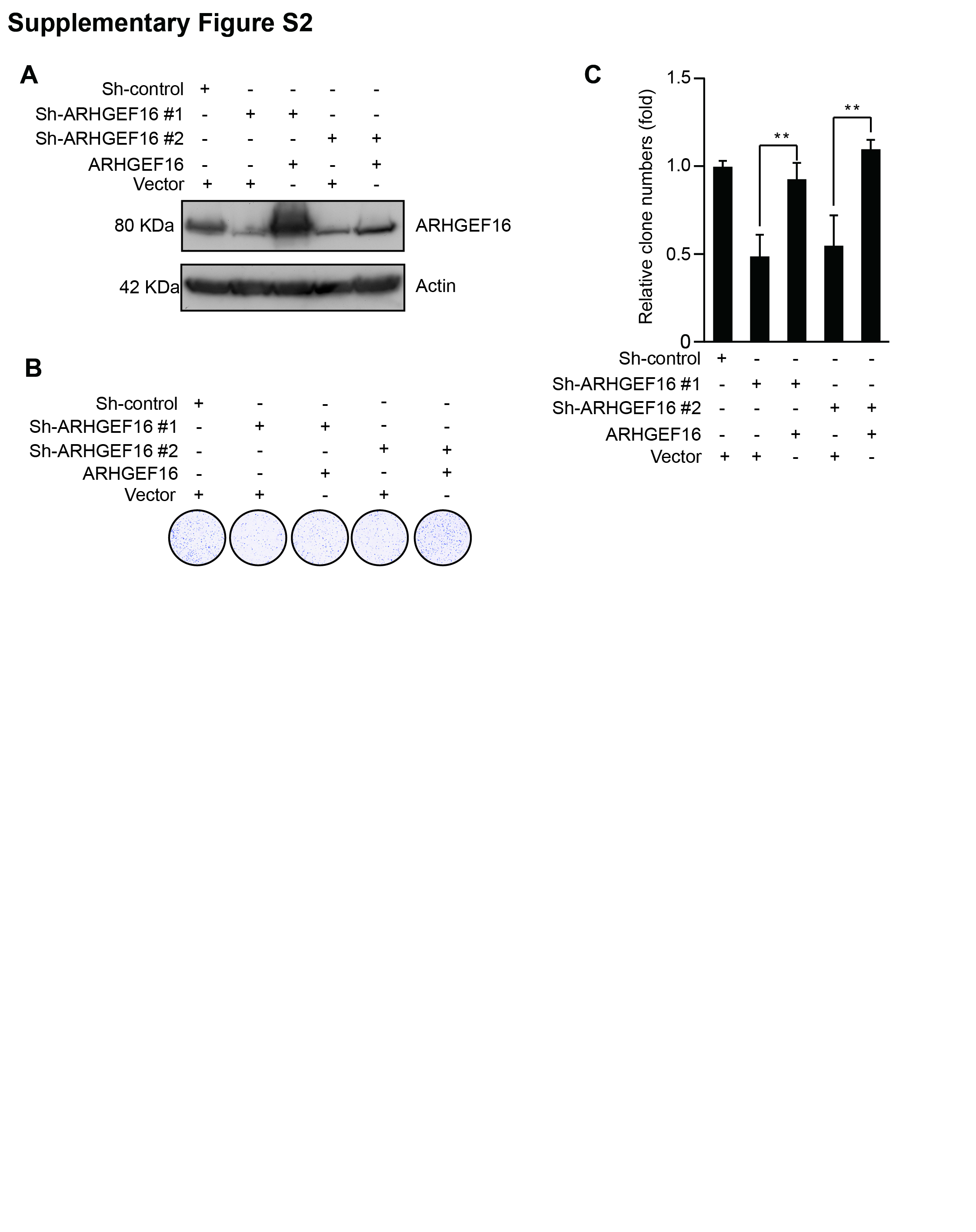

Supplement: Supplementary file 2 — Supplementary Figure S2 [file 41419_2020_2830_MOESM2_ESM.tif]

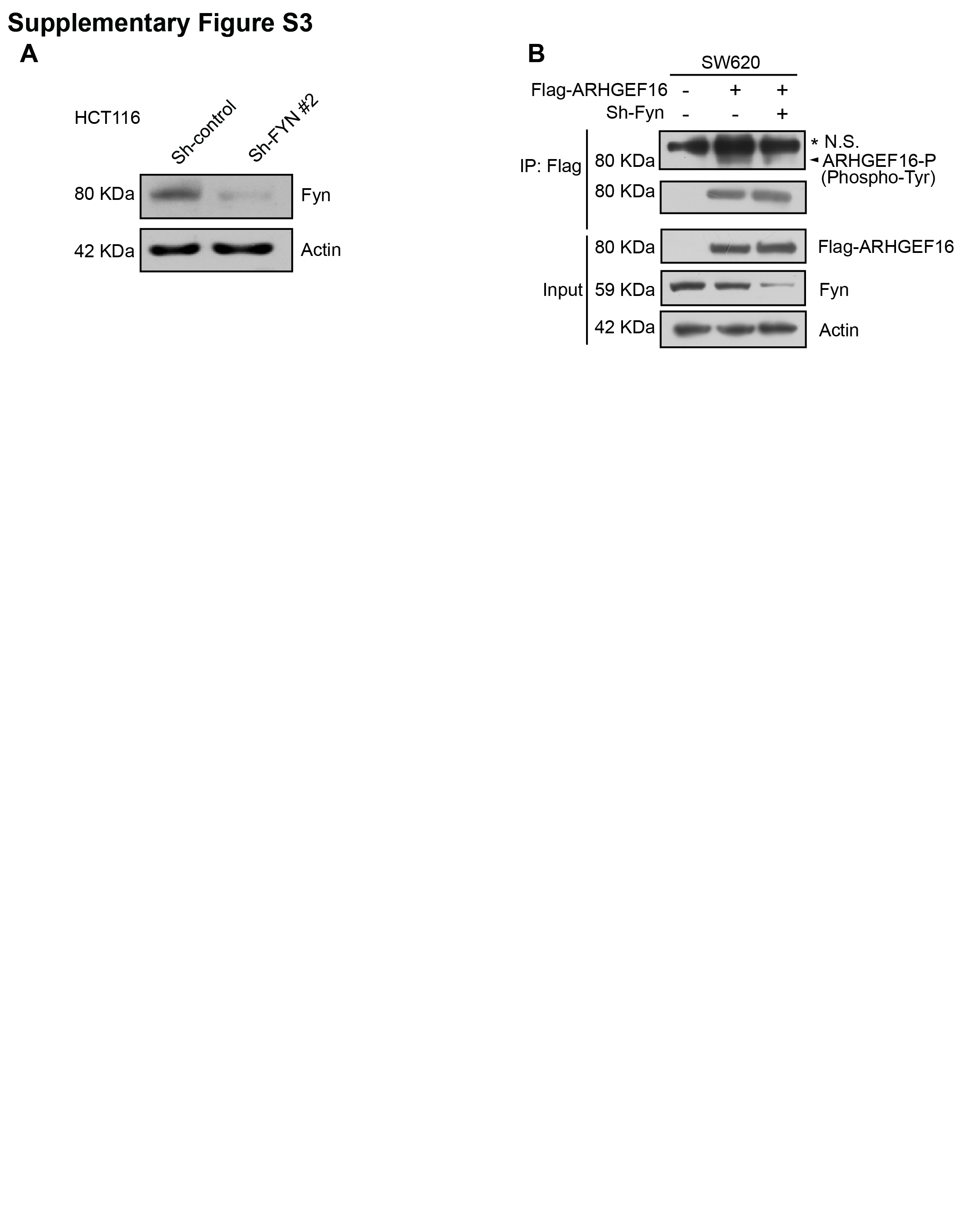

Supplement: Supplementary file 3 — Supplementary Figure S3 [file 41419_2020_2830_MOESM3_ESM.tif]

**Fig. 3c**

**HCT116 tumors**

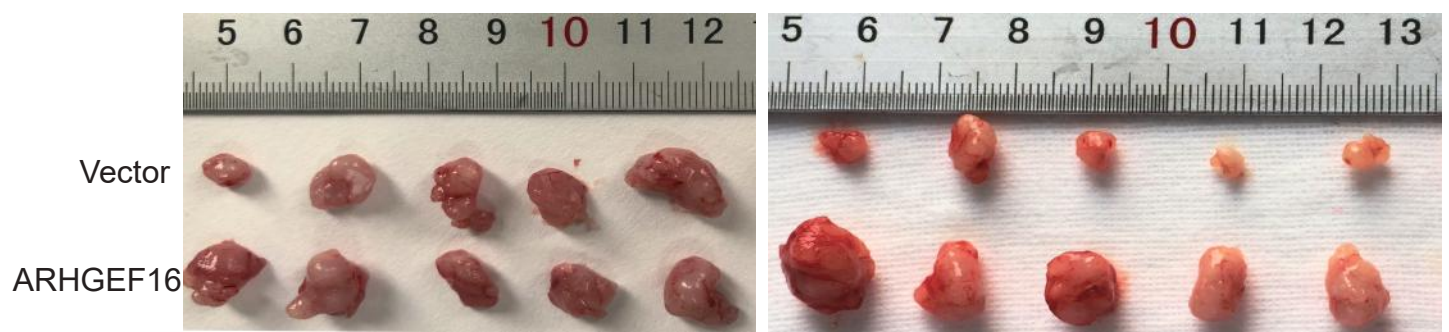

Supplement: Supplementary file 7 — HCT116 tumors [file 41419_2020_2830_MOESM7_ESM.pdf]
