# Supplementary material for: FYN is required for ARHGEF16 to promote proliferation and migration in colon cancer cells
Source: Cell Death Dis. 2020 Aug 7;11(8):652. doi: 10.1038/s41419-020-02830-1 (PMC7435200; doi:10.1038/s41419-020-02830-1)
Supplement: Supplementary file 4 — Supplementary Figure Legends [file 41419_2020_2830_MOESM4_ESM.docx]

**Supplementary Figure S1 ARHGEF16 increases colorectal cancer cell proliferation. a** SW620 cells were transfected with Sh-control or Sh-ARHGEF16 #2 for 48 h and harvested for WB analysis with the indicated antibodies. **b** HT29 cells were transfected with Sh-control or Sh-ARHGEF16 #1 for 48 h and harvested for WB analysis with the indicated antibodies. **c** HT29 cells were transfected with Vector or Lv-ARHGEF16 for 48 h and harvested for WB analysis with the indicated antibodies. **d** ARHGEF16 overexpression increased the colony formation ability of SW480 cells. **e** Quantification of the colony formation rates shown in Supplementary Figure S1D. *P*-values were obtained by the two-side Students *t*-test. Data are shown as the mean ± SD (n = 5). ***P* < 0.01. **f** ARHGEF16 overexpression increased the proliferation rate of SW480 cells, as shown by Edu staining. **g** Quantification of the formation rates as shown in Fig. 2k. *P*-values were obtained by the two-side Students *t*-test. Data are shown as the mean ± SD (n = 5). **P* < 0.05. **h** Nude mice were injected subcutaneously with 1.0×10^7^ cells/mouse for each of the indicated stable SW480-ARHGEF16 cell lines. Results are presented for isolated tumors**. i** tumor weight in nude mice were injected subcutaneously with the indicated stable SW480-ARHGEF16 cell lines. *P*-values were obtained by the two-side Students *t*-test. Data are shown as the mean ± SD (n = 6). ***P* < 0.01. **j** Overexpression of ARHGEF16 in xenografts was confirmed by WB analysis.

**Supplementary Figure S2 Exogenous** **ARHGEF16 could rescued inhibition of cell proliferation by ARHGEF16 knockdown. a** WB analysis was used to detect ARHGEF16 in HCT116 cells in which ARHGEF16 is knockdown. **b, c** ARHGEF16 knockdown with Sh-control or Sh-ARHGEF16 #1 and Sh-ARHGEF16 #2 in SW620 cells, then the cells were transfected with Vector or Lv-ARHGEF16. ***P* < 0.01. *P*-values were obtained by the two-side Students *t*-test. Data are shown as the mean ± SD (n = 5).

**Supplementary Figure S3** WB analysis of ARHGEF16-p and FYN in colon cancer cells. **a** HCT116 cells were transfected with Sh-control or Sh-FYN #2 for 48 h and harvested for WB analysis with the indicated antibodies. **b** Cell lysates from SW620 cells transfected with the indicated plasmids were immunoprecipitated with an anti-Flag antibody and total cell lysates were analyzed with antibodies against Flag, FYN and phosphor-Tyr. N.S. not significant.
