# Supplementary material for: FYN is required for ARHGEF16 to promote proliferation and migration in colon cancer cells
Source: Cell Death Dis. 2020 Aug 7;11(8):652. doi: 10.1038/s41419-020-02830-1 (PMC7435200; doi:10.1038/s41419-020-02830-1)
Supplement: Supplementary file 6 — Supplementary Tables [file 41419_2020_2830_MOESM6_ESM.docx]

**Table S1. Target sequences of gene-silencing constructs**

| Name | Sequence Source |
| --- | --- |
| sh-ARHGEF16 #1 | 5’-AGGACTTTGCCCGCTTCATCA-3’ (Huang, 2018) |
| sh-ARHGEF16 #2 | 5’- GGAGGCACCAATAGCGATTAT-3 (Hiramoto-Yamaki, 2010) |
| sh-FYN #1 | 5’-AAGACATGTGGTGTATATAAA-3’ (Jia, 2012) |
| sh-FYN #2 | 5’- GGACTCACCGTCTTTGGAG-3’ This study |

**Table S2. Primer sequences for qPCR**

| Primer name | Sequence |
| --- | --- |
| ARHGEF16- Forward | 5’-GATACGCTCTGCCTCAAGAC-3’ |
| ARHGEF16-Reverse | 5’-GGGACTTGACCTTGCTGAAG-3’ |
| FYN-Forward | 5’-tgtcacaaagggatgccaag-3’ |
| FYN-Reverse | 5’-ttccccaaactgcccatttc-3’ |
| GAPDH-Forward: | 5’-CAGGGCTGCTTTTAACTCTG-3’ |
| GAPDH-Reverse | 5’-GATTTTGGAGGGATCTCGC-3’ |

**Reference**

1. Hiramoto-Yamaki, N. *et al.* Ephexin4 and EphA2 mediate cell migration through a RhoG-dependent mechanism. *The Journal of Cell Biology*. **190**, 461-477 (2010).
2. Huang, D. *et al.* GLI2 promotes cell proliferation and migration through transcriptional activation of ARHGEF16 in human glioma cells. *J Exp Clin Cancer Res*. **37**, 247 (2018).
3. Jia, Rui, *et al*. The N-terminal region of IFITM3 modulates its antiviral activity by regulating IFITM3 cellular localization. *Journal of virology* **86,** 13697-13707 (2012).
